# Factors Affecting Influenza Vaccination Uptake and Attitudes among Lebanese University Students: The Impact of Vaccination Promotional Programs and COVID-19 Pandemic

**DOI:** 10.3390/vaccines11050949

**Published:** 2023-05-05

**Authors:** Nisreen Mourad, Lidia Mourad, Dalal Hammoudi Halat, Zeina Farah, Mohamed Hendaus, Israa El Sayed Trad, Marwan El Akel, Jihan Safwan, Mohamad Rahal, Samar Younes

**Affiliations:** 1 School of Pharmacy, Lebanese International University, Lebanon; 2 INSPECT-LB: Institut National de Santé Publique, Épidémiologie Clinique et Toxicologie-Liban, Beirut 1103, Lebanon; 3 Academic Quality Department, QU Health, Qatar University, Doha 2713, Qatar; 4 Epidemiological Surveillance Program, Ministry of Public Health, Beirut, Lebanon; 5 Global Health Institute Department, American University of Beirut, Beirut 11-0236, Lebanon; 6 School of Education, Lebanese International University, Beirut 14404, Lebanon; 7 International Pharmaceutical Federation, 2517 JP The Hague, The Netherlands

**Keywords:** influenza, vaccines, university students, vaccine hesitancy, uptake and attitudes, COVID-19, vaccination promotional programs

## Abstract

Vaccination is the most effective preventative strategy against influenza, yet university students’ influenza vaccination uptake remains low. This study aimed firstly to determine the percentage of university students who were vaccinated for the 2015–2016 influenza season and to identify reasons for non-vaccination, and secondly to examine the impact of external factors (on-campus/online influenza awareness campaigns and COVID-19 pandemic) on their influenza vaccination uptake and attitudes for the 2017–2018 and 2021–2022 influenza seasons. A descriptive study was conducted over three phases for three influenza seasons at a Lebanese university in the Bekaa Region. Based on data collected in 2015–2016, promotional activities were developed and implemented for the other influenza seasons. This study was conducted using an anonymous, self-administered questionnaire by students. The majority of the respondents in the three studies did not receive the influenza vaccine (89.2% in the 2015–2016 study, 87.3% in the 2017–2018 study, and 84.7% in the 2021–2022 study). Among the unvaccinated respondents, the main reason for non-vaccination was that they thought that they did not need it. The primary reason for vaccination among those who were vaccinated was that they believed they were at risk of catching influenza in a 2017–2018 study and due to the COVID-19 pandemic in the 2021–2022 study. As for attitudes towards influenza vaccination post-COVID-19, significant differences were shown among the vaccinated and unvaccinated respondents. The vaccination rates among university students remained low despite of the awareness campaigns and COVID-19 pandemic.

## 1. Introduction

Influenza is a highly contagious respiratory viral infection that remains a major cause of morbidity and mortality worldwide along with substantial economic burden. According to the World Health Organization (WHO), influenza affects one-billion of the world’s population each year, resulting in about three- to five-million cases of severe illness and about 290,000 to 650,000 influenza-related respiratory deaths [1]. Annually, in the United States alone, influenza is estimated to cause a total economic burden of $11.2 billion ($6.3–25.3 billion) [2]. Influenza complications can occur mostly among high-risk individuals such as children, the elderly, pregnant and postpartum women up to two weeks after delivery, those with underlying medical conditions, people who live in nursing homes, and certain racial and ethnic groups [3]. However, influenza can affect anyone including university students, whom, once infected, become a source for the transmission of the disease while suffering from absenteeism and loss of productivity themselves [4].

Vaccination is the most effective preventative strategy against influenza [4]. Despite the fact that annual influenza vaccination is particularly recommended for individuals who are at high-risk of influenza complications, as well as for those who live with or care for them, it is important to note that healthy adults can also benefit from the protection provided by the influenza vaccine [4]. In general, university students’ influenza vaccination uptake remains low globally although they are at risk of acquiring and spreading the infection contributing to the burden of disease [5,6]. The reasons for university students’ influenza vaccine hesitancy are complex, but can fall under complacency, lack of confidence, and inconvenience, which were identified by the WHO as key reasons for vaccine hesitancy [7]. Reported barriers to influenza vaccination included low perception of the personal risk and severity of influenza, doubting vaccine efficacy, vaccination costs and accessibility, and safety concerns [5,8,9,10]. On the other hand, key drivers for vaccination were mainly self-protection, protection of others by prevention of transmission, belief in vaccine effectiveness, and encouragement from a medical professional [8,9,10,11].

It is important to note that major barriers to influenza vaccination, in addition to the prevalence of misinformation among university students, should be addressed [12]. Thus, influenza vaccination enhancement programs such as campus vaccination awareness campaigns should be tailored to target specifically university students’ barriers to, and facilitators and misconceptions of, vaccination [13]. In fact, targeted and coordinated education and activities delivered through multiple communication strategies have been shown to reduce vaccine hesitancy, mitigate vaccine misinformation, and promote vaccination uptake [10,12]. All these efforts lead to improved perceptions and attitudes towards, and practices of, vaccination, ultimately resulting in an increase in university-wide influenza vaccination. There is, however, limited knowledge on vaccination programs that might successfully increase university students’ seasonal influenza vaccination rates [6], although, recently, social marketing interventions or programs have been found effective in addressing vaccine hesitancy and improving seasonal influenza vaccination among young adults [6,14].

Furthermore, external factors can also influence influenza vaccine uptake, where recent reports have shown that the COVID-19 pandemic had positively impacted the vaccination rates [15]. In fact, an improved health-seeking behavior has been noticed with a change in the health risk perception and attitudes towards vaccinations [15,16]. Recently, a meta-analysis found that the COVID-19 pandemic has resulted in an increased intention for influenza vaccination globally, irrespective of region, age, gender, and occupation [15]. This intention has led to an evident increase in influenza vaccination uptake [10].

The drivers of and barriers to influenza vaccine acceptance among Lebanese university students have not been fully explored, and the impact of vaccination promotional programs and COVID-19 pandemic on influenza vaccine uptake is an area of emerging interest. Thus, by identifying the drivers and barriers to vaccine acceptance and evaluating the effectiveness of different vaccination promotional strategies and natural events, our study will provide evidence-based information on effective techniques that aim to improve future influenza vaccination programs targeting this population, thus yielding an increase in influenza vaccination rates and reduction in the influenza outbreaks and their consequences.

As such, this study aimed, firstly, to determine the percentage of university students who were vaccinated for the 2015–2016 influenza season and to identify reasons for non-vaccination among those who were not. Secondly, it aimed to examine the impact of external interventions on influenza vaccination uptake and attitudes among university students, initially through an on-campus influenza awareness campaign for the 2017–2018 influenza season, and then through a web-based influenza awareness campaign in addition to the COVID-19 pandemic for the 2021–2022 influenza season. Moreover, the study intended to explore the demographic and socioeconomic factors that affected university students’ vaccine uptake, and to assess the COVID-19 vaccine uptake for those who received the influenza vaccine for the 2021–2022 influenza season.

## 2. Materials and Methods

### 2.1. Study Design

A descriptive study was conducted over three phases, from January–February 2016 (for the 2015–2016 influenza season), December 2017 (for the 2017–2018 influenza season), and January–May 2022 (for the 2021–2022 influenza season) at a Lebanese university in the Bekaa Region that extends over an area of 400,000 square meters. This university encompasses five schools (School of Arts and Science, School of Business, School of Education, School of Engineering, and School of Pharmacy) in addition to a Freshman Program, with a total number of students of approximately 3000, 4000, and 5000 for the 2015–2016, 2017–2018, and 2021–2022 academic years, respectively.

Based on the data collected in 2015–2016, promotional activities were developed and implemented for the 2017–2018 and 2021–2022 influenza seasons by the study team. Thus, prior to the 2017–2018 study, an awareness campaign entitled “Beat the Flu” was conducted between October 9 and 12, 2017 at the university campus in which 416 students participated, out of which 385 were unvaccinated. The campaign focused on influenza vaccination, during which information concerning influenza’s signs and symptoms, methods of transmission, complications, and preventative measures was provided with thorough elaboration about vaccination. Educational material such as informative leaflets and fact sheets were handed out, and data regarding students’ vaccination status were collected. Of note is that all the campaign materials were culturally sensitive and easy to read and understand, with clear and concise language, short sentences, and simple graphics intended to make the materials visually appealing and further aid in understanding. All of these measures were taken to ensure that participants were able to fully comprehend and engage with the campaign content, regardless of their health literacy levels. In addition, the campaign’s advertisements were designed to be highly attractive and engaging with catchy phrases and visually appealing graphics to capture the attention of students and encourage their engagement with the campaign.

Preceding the 2021–2022 study, a continuation of the “Beat the Flu” campaign was carried out online during October 2021, using various social media platforms including Facebook and Instagram. Moreover, the campaign material was sent to the students’ emails to ensure its delivery to all. In addition to the information covered by the previous campaign, the 2021 campaign included the importance of influenza vaccination during the COVID-19 pandemic. Additionally, similar to the previous campaign, this campaign’s educational material and advertisements were characterized by their simplicity, comprehensibility, cultural sensitivity, and attractiveness.

**Figure 1:**
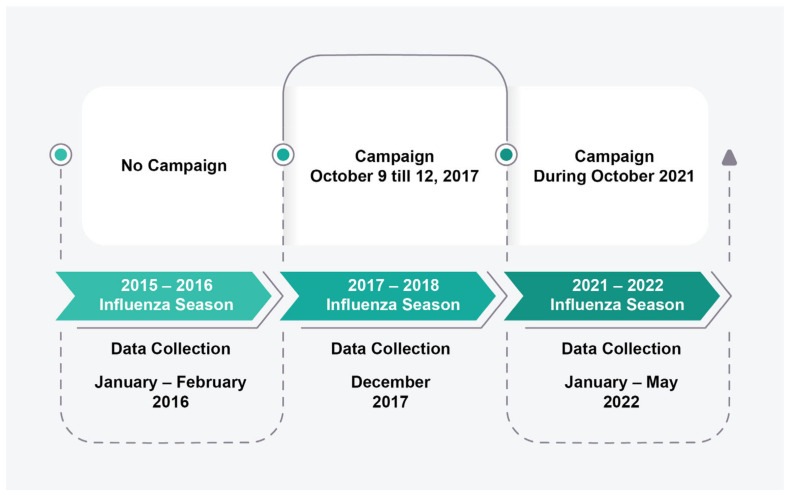
Timeline for the study’s three phases.

The timeline of this study, showing the three influenza seasons targeted, the awareness campaigns carried out, and the timeframe of data collection are depicted in Figure 1.

### 2.2. Sample Size Calculation

With reference to Epi-info software, a minimum sample of 343 participants for the 2015–2016 study and 358 participants for the 2021–2022 study was needed, the expected frequency was kept at 50% to yield the largest minimal sample size possible to allow for adequate power for statistical analysis and produce a 95% confidence interval with an acceptable margin of error of 5%. For the 2017–2018 study, it tackled the unvaccinated campaign participants (385), which constituted 10% of the campus population; accordingly, a minimum sample of 138 participants for this study was required to produce a 95% confidence interval with an acceptable margin of error of 5%.

### 2.3. Variables

This study was conducted using a questionnaire prepared by the research team and was updated before each administration to address all research questions of each phase. To ensure validity, each time the questionnaire was pre-tested and evaluated by the researchers to ensure clarity of the questions, and comments were integrated in the final versions of the questionnaire. In general, the questionnaire was divided into two sections: (1) demographic characteristics, and (2) vaccination status and reasons for non-vaccination. The 2017–2018 and 2021–2022 studies included an addition of reasons for vaccination, whereas, in the 2021–2022 study, a section related to the effect of COVID-19 on influenza vaccination was added.

### 2.4. Outcomes Measured

The 2015–2016 study aimed to determine the percentage of university students who were vaccinated for that year’s influenza season and to identify reasons for non-vaccination among those who were not in order to guide future vaccination intervention programs. In order to investigate how external factors influenced the university students’ influenza vaccination uptake and attitudes, two additional studies were conducted for the 2017–2018 and 2021–2022 influenza seasons.

The 2017–2018 study intended to determine the impact of an on-campus influenza awareness campaign, whereas the 2021–2022 study examined the effect of a web-based influenza awareness campaign in addition to the COVID-19 pandemic. Both studies assessed these factor’s influence on the uptake of and attitudes toward influenza vaccination for the unvaccinated campaign’s participants for the 2017–2018 study and for all that university’s students for the 2021–2022 study. Of note is that to measure the effectiveness of both campaigns, following them respondents were asked about their reasons for vaccination, and one of the options provided was the “Beat the Flu” campaign. As such, we assessed the percentage of respondents who selected this option as an indication of the campaign’s effectiveness.

In addition, the demographic and socioeconomic factors that characterize university students’ vaccination uptake were explored.

### 2.5. Study Population

In the 2015–2016 study, an anonymous, paper-based questionnaire was distributed to all students at a Lebanese university in the Bekaa Region, whereas, for the 2017–2018 study, an anonymous, web-based questionnaire was sent to the unvaccinated campaign participants on the same campus. As for the 2021–2022 study, an anonymous, web-based questionnaire was sent to all students on the same campus as well. In each questionnaire version, the study’s aims were explained to students who filled in the anonymous, self-administered questionnaire.

### 2.6. Data Analysis

Collected data were cleaned, encoded, and then analyzed; categorical data were reported as frequencies and percentages; Chi-square test and Fisher’s exact test were used to test the association between vaccination status and the different study variables. Data were analyzed using R software version 4.2.2 and R studio version 2022.2.3.492. A *p*-value of less than 0.05 was considered statistically significant.

### 2.7. Ethical Considerations

The study was approved by the Lebanese International University Research Committee. Participants’ privacy, anonymity, and confidentiality were protected through using codes, limiting access of data to the study team, and securely storing data. In addition, students who agreed to participate provided informed consent.

## 3. Results

There were a total of 1023 respondents in the 2015–2016 study, 126 in the 2017–2018 study, and 1016 in the 2021–2022 study.

### 3.1. Demographic Characteristics

#### 3.1.1. The 2015–2016 Study

In the study conducted in 2015–2016 (*n* = 1023), the majority of the respondents (89.2%) did not receive the influenza vaccine for that year, 64.3% were aged between 20 and 29 years, 64.2% were females, 84% were Lebanese, and 95.2% were unmarried. As for the lifestyle of the participants, it was noted that out of the current smokers (16.9%), only 8.1% had received the vaccine. An income of more than $1000 per month was reported by 16.4%, yet only 13.7% of this 16.4% had their influenza shots. The students who responded to the questionnaire were from all majors; among those, 18.1% were pharmacy students, of whom only 11.9% were vaccinated.

#### 3.1.2. The 2017–2018 Study

The majority of respondents (87.3%) in the study from 2017–2018 (*n* = 126) had not received the influenza shot despite the “Beat the Flu Campaign”. The majority of participants (67.5%) were aged between 20 and 29 years, 81% were females, 87.3% were Lebanese, and 94.4% were unmarried. Regarding the participants’ lifestyle, it was noticed that among the current smokers (12.7%), just 18.8% had received the vaccination. About 30.2% of individuals reported having a household income of above $1000 per month, and 10.5% of those people had their influenza vaccines. Among the respondents who were from all majors, 67.5% students were majoring in health-related fields such as pharmacy, of whom only 14.1% were vaccinated.

#### 3.1.3. The 2021–2022 Study 

In spite of the web-based “Beat the Flu” campaign and the presence of the COVID-19 pandemic, still the majority of respondents (84.7%) in the study performed in 2021–2022 (*n* = 1016) did not receive the influenza vaccine. The majority of respondents (60.5%) were aged between 20 and 29 years, 72.3% were females, 75.4% were Lebanese, and 93.1% were unmarried. Regarding the smoking status, it was noticed that just 16.2% of the 23.1% current smokers had taken the vaccination. A total of 29.2% participants reported having a monthly income of above $1000, yet only 16.8% of them had received the influenza vaccine. All majors were represented among the respondents, and of those who were studying pharmacy (34.3%), only 12.1% had had their vaccination.

Demographic characteristics for the three studies are detailed in Table 1.

### 3.2. Vaccine Uptake and Reasons for Hesitancy

The majority of the vaccinated respondents in all the studies reported that they received the influenza vaccine at the pharmacy (78.2% in the 2015–2016 study, 81.3% in the 2017–2018 study, and 21.3% in the 2021–2022 study).

Our analysis compared seven perceptions of hesitancy to influenza vaccine uptake among the non-vaccinated individuals (89.2% in the 2015–2016 study, 87.3% in the 2017–2018 study, and 84.7% in the 2021–2022 study) (Table 2). The majority reported that the main reason for not getting vaccinated was that they believed that they did not need it (42.7% in the 2015–2016 study, 40.9% in the 2017–2018 study, and 36.6% in the 2021–2022 study), while the belief that the vaccine was not beneficial was reported by 17.5% in the 2015–2016 study, 20.9% in the 2017–2018 study, and 15.6% in the 2021–2022 study. What is noteworthy is that some participants stated that a doctor/pharmacist did not recommend the vaccination (17.2% in the 2015–2016 study, 10% in the 2017–2018 study, and 15.2% in the 2021–2022 study), while a considerable number conveyed that they do not like needles (15.3% in the 2015–2016 study, 24.5% in the 2017–2018 study, and 10.9% in the 2021–2022 study). However, the lowest-ranked barrier was “it costs too much” (1.9% in the 2015–2016 study, 0.9% in the 2017–2018 study, and 6.3% in the 2021–2022 study).

Eight perceptions on influenza vaccination uptake were explored in the 2017–2018 and 2021–2022 studies, and the presence of the COVID-19 pandemic was added to the 2021–2022 study (Table 2). In the 2017–2018 study, the majority of the respondents reported taking the vaccine because they believed that they are at risk of catching it (75%), that the influenza vaccine is effective (50%), and that it is generally safe (31.3%). Around a quarter of the respondents (25%) reported that they took the influenza vaccine because they were informed about it through the “Beat the Flu” campaign.

In the 2021–2022 study, the presence of the COVID-19 pandemic encouraged 61.3% of the respondents to take the vaccine, and the intention to lessen the virus transmission and the belief of vaccine safety both ranked second with 36.1%. Approximately a quarter of the participants agreed that the vaccine is effective (27.7%) and that they are at risk of catching it (27.1%). The belief that influenza is a serious disease was reported by 18.1% and encouragement by others to take the influenza vaccine was stated by 12.3%. The “Beat the Flu” campaign was a source of encouragement to take the vaccine in only 11% of the respondents.

Table 2 details the respondents’ characteristics in relation to the location where they received the vaccine as well as their reasons for receiving or refusing the influenza vaccine.

### 3.3. Attitudes towards Influenza Vaccination Post-COVID-19 in the 2021–2022 Study

Following the COVID-19 pandemic, a positive attitude and higher uptake of the vaccine was reported among the vaccinated respondents who agreed with the statements “taking the flu vaccine is important in order not to mix flu symptoms with COVID-19 symptoms” (32.3% vaccinated vs. 21.0% unvaccinated; *p* = 0.0029) and “vaccines are important to avoid pandemics” (47.1% vaccinated vs. 37.2% unvaccinated; *p* = 0.025). However, no statistical difference was found between respondents who agreed with the statements “vaccines are effective and crucial to guaranteeing public health” (42.6% vaccinated vs. 35.4% unvaccinated; *p* = 0.107) as well as “vaccines are a fraud designed to profit pharmaceutical companies” (7.7% vaccinated vs. 6.0% unvaccinated; *p* = 0.107), implying a lack of effect on their attitude. As for those who reported no change with their attitude, most of them were unvaccinated (11.0% vaccinated vs. 35.4% unvaccinated; *p* < 0.0001) and a statistical difference was noted showing that COVID-19 did not encourage them to receive the influenza vaccine. On another note, the uptake of the COVID-19 vaccine was reported by respondents, whereby two doses of the COVID-19 vaccine had been received by 69.7% of those who received their influenza vaccine and 57.1% of those who did not.

Table 3 shows the impact of COVID-19 on influenza vaccination in the 2021–2022 study.

## 4. Discussion

To our knowledge, this study is the first in Lebanon to explore vaccination rates and attitudes towards influenza vaccination among university students in three influenza seasons, using interventional awareness and descriptive surveys. In Lebanon, which has a burden of laboratory-confirmed influenza of about 14% and mortality of 3.8% [17], and with crises severely impacting the once top-tier healthcare system [18], an investigation of influenza vaccination status is important. In 2022, a meta-analysis of global influenza vaccination recommended more studies on this topic from the Eastern Mediterranean region [19], indicating that a profound understanding of influenza vaccination uptake and attitudes from our area is critical.

According to our data, the vaccination rates in 2015–2016, 2017–2018 and 2021–2022 studies remained low, at 10.8%, 12.7%, and 15.3%, respectively. These rates are lower than a previous rate of close to 28% in a Lebanese report in 2015 [20], although lower rates were reported in nearby countries, including Turkey [21] and Saudi Arabia [22], with influenza vaccination reported in 8% and 9% of the population, respectively. In 2021–2022, influenza vaccine uptake was the highest among the three studies. This may have been affected by the online awareness campaign, “Beat the Flu”, addressed to students, at a time when social media use and remote interaction was at its peak due to successive pandemic waves hitting Lebanon [23]. In this season, about 11% of the participants mentioned the campaign as a reason to receive the vaccine. Recently, social media users were reported to be more likely to receive the vaccinated against influenza [24]. Social media platforms may have the potential to publicize influenza vaccine information, and may encourage users to get vaccinated annually. Another factor that may explain the higher influenza vaccination rate in the 2021–2022 study may be the ability of the COVID-19 pandemic to renew awareness of respiratory infections, primarily influenza, an observation highly probable and widely reported in the literature [25,26,27,28], with popuar perceptions of the influenza vaccine during COVID-19 pandemic having positive impact on the influenza vaccine [29]. Specifically, the university health committee, established early during COVID-19, may have exercised a specific influence on university students by extensive awareness campaigns on COVID-19 [30], but this remains inconclusive and cannot be directly assessed using our results. In Lebanon, Youssef et al. reported that influenza vaccine intake in the 2021–2022 season was higher than the previous season among healthcare workers [31], a finding consistent with our results. By contrast, the COVID-19 pandemic has increased the hesitancy towards influenza vaccination in a study from Saudi Arabia [32]. With the world emerging from COVID-19 and its influence fading, more awareness is needed to underpin the importance of influenza vaccination.

Upon comparing the demographic data of our participants across the three years, no major statistically significant differences were observed between the vaccinated and non-vaccinated population, and this was true for independent variables including age, nationality, study major, marital status, smoking, alcohol consumption, or comorbidities. Accordingly, these variables cannot be presumed to influence the status of the influenza vaccination. Yet, the female gender was associated with a lower probability of influenza vaccination in the 2021–2022 study, in contrast to other studies [33]. Furthermore, in the 2017–2018 study, participants who did not know or respond to the family monthly income were more likely to be vaccinated. In a very recent study, adults with a lower total family income had at least 20% decreased odds of receiving the influenza vaccine [34]. Hence, influenza vaccination may be significantly impacted by income, and increased influenza vaccination rates among persons with lower incomes has to be prioritized from a general public health perspective.

The percentage of vaccinated students among the three studies ranged between 11 and 15%, lower than those reported among students elsewhere, especially in health majors [35,36]. Such low influenza vaccination rates among these students is disturbing, and may add to data showing that knowledge of the vaccine does not necessarily ensure its uptake or behavioral change [37]. Reinforcing the importance of vaccination and providing additional information targeted to health students may be needed to raise vaccination rates in this population. This is important considering the future contact between this group of future healthcare workers and patients.

In the three studies, an average of 60% of participants reported receiving the influenza vaccine at a pharmacy, with lower percentages reporting receiving it at another health facility. In a systematic literature review and meta-analysis of influenza vaccination acceptance, the vaccination rate was 24% higher in those who used the pharmacy-based vaccination [38]. Pharmacists are regarded as professional figures in the health sector, qualified to improve social accountability and confirmed to have a central role in the promotion of vaccination [39]. As such, their involvement in immunization, whether as educators, facilitators, or administrators of vaccines, resulted in increased vaccine uptake [40]. Capitalizing on these data about pharmacists’ role in vaccination, it may be reasoned that pharmacist intervention may increase, and further studies in this regard involving Lebanese pharmacists may be interesting.

Some reasons for influenza vaccine hesitancy were consistent across the three studies, namely the beliefs that the vaccine was not needed or not beneficial. In a study by Davis and colleagues [41], the most common reason for not receiving the influenza vaccine was not being concerned about the infection. In fact, the substantial healthcare and economic burden resulting from influenza was estimated at an annual sum reaching 25 billion US dollars [2]. As individuals may not be well aware of such a burden, raising awareness and dissemination of knowledge regarding influenza and the key role of vaccination are crucial, especially to the population of university students. Fear of needles, as well as the fear from the risk of becoming sick from the vaccine, were also reasons to avoid it among our participants, and this is in parallel with results reported previously [13,41,42,43]. Approximately 31% and 36% of vaccinated participants in the 2017–2018 and 2021–2022 studies, respectively, believed that the vaccine is generally safe. Hence, with a rough one-third of the studied population only being convinced about vaccine safety, continuous updates about the safety of the vaccine are recommended strategies to reduce vaccine hesitancy. Moreover, the cost of the influenza vaccine remains an issue, and the proportion of participants mentioning this as a reason for not taking the vaccine increased by about 3–6 folds in 2021–2022 compared to the previous two seasons when the questionnaires were administered. This may be explained by the monetary devaluation and the serious economic crisis that is affecting Lebanon as of 2019, and which had exerted its effects on costs of healthcare, including vaccines [44]. Likewise, the barrier of vaccine unavailability increased in 2021–2022, perhaps due to drug shortages in Lebanon amidst the crisis [45]. In fact, reports about cost barriers to influenza vaccine exist even in the developed world [43]. This emphasizes the need for convenient and affordable access to influenza vaccines to overcome financial barriers. Explicating and targeting such culprits promise to improve vaccination rates. It is peculiar that some participants reported that a doctor/pharmacist did not recommend the influenza vaccination, despite the scientific consensus on the vaccine’s importance [46]. The reason for such reporting cannot be fully explained, and warrants further investigation, as annual influenza vaccination should be recommended for individuals from 6 months and above without contraindications.

In the 2021–2022 study, and in addition to the general positive impact the pandemic had on influenza vaccination perceptions, and higher influenza vaccination rate among those who received the COVID-19 vaccine, a statistical significance of the stated need to vaccinate to avoid mixing influenza and COVID-19 symptoms and the importance of vaccines in preventing pandemics was observed between vaccinated and non-vaccinated participants. Statistically, vaccinated participants were more likely to believe in these two statements, suggesting that fear and uncertainty emerging from the pandemic may have encouraged different health practices. The pandemic has yielded lifestyle changes, mutated the way individuals think, remodeled the delivery of healthcare, and propagated a sense of vulnerability [47,48,49]. This highlights the importance of providing resources for education about COVID-19 and respiratory infections in general as a measure to increase vaccination rate. As for participants declaring that their attitudes towards influenza vaccination did not change post COVID-19, these were statistically less likely to be vaccinated, indicating that their vaccine hesitancy was not affected by the pandemic, indicating the need for additional triage and counselling.

The strengths of this study lie in its multimodal approach and targeting different cohorts of university students, thus allowing us to capture variations in the drivers and barriers to influenza vaccine across years, and to include the influence of the COVID-19 pandemic on the influenza vaccine status and its perceptions among this population. This information is crucial for public health practitioners and policy-makers to design targeted and effective influenza vaccination programs that are tailored to the specific needs and concerns of this population and responsive to the unique challenges and circumstances posed by the COVID-19 pandemic, all of which leads to increased vaccination rates and reduced influenza outbreaks. It also exposes the prototype of two different awareness campaigns, both face-to-face and virtual, on knowledge and perceptions towards the influenza vaccine. This is important because awareness campaigns are a key strategy for promoting vaccine uptake, and our study provides evidence-based information on the most effective approaches for reaching and engaging university students and taking advantage of different communication channels. However, the study does have limitations; the response rate was not equal across our three studies, where the 2017–2018 study did not reach the minimum required sample, and this may have affected our results. The administration routes of the questionnaire were different, and this may have induced some bias. In addition, although we acknowledge the method we implemented for measuring the campaigns’ effectiveness may have limitations, we believe that this approach provides valuable insight into the impact of both campaigns. Moreover, the data were collected from a single university and may not reflect a clear image of different university students all over Lebanon. Further studies addressing influenza vaccine uptake and attitudes among university students on a national level are recommended.

## 5. Conclusions

The vaccination rates among university students remained low despite the vaccination promotional programs and the COVID-19 pandemic. This necessitates the implementation of additional targeted, evidence-based interventions to reshape the students’ current perspective on influenza vaccination and that could ultimately lead to long-term changes among university students’ attitudes.

## Figures and Tables

**Table 1:** Comparison of demographic characteristics between vaccinated and not-vaccinated respondents for the 2015–2016, 2017–2018, and 2021–2022 studies.

	2015–2016			2017–2018			2021–2022		
Characteristics	Vaccinated (N = 110)	Not-Vaccinated (N = 913)	*p*-Value	Vaccinated (N = 16)	Not-Vaccinated (N = 110)	*p*-Value	Vaccinated (N = 155)	Not-Vaccinated (N = 861)	*p*-Value
	*n* (%)	*n* (%)		*n* (%)	*n* (%)		*n* (%)	*n* (%)	
**Age**									
16–19	40 (36.4)	323 (35.4)		7 (43.8)	34 (30.9)		52 (33.5)	309 (35.9)	
20–29	70 (63.6)	588 (64.4)	0.867	9 (56.2)	76 (69.1)	0.460	92 (59.4)	523 (60.7)	0.111
30–39	0 (0.0)	2 (0.2)		0 (0.0)	0 (0.0)		0 (0.0)	3 (0.3)	
40–49	0 (0.0)	0 (0.0)		0 (0.0)	0 (0.0)		0 (0.0)	0 (0.0)	
≥50	0 (0.0)	0 (0.0)		0 (0.0)	0 (0.0)		11 (7.1)	26 (3.0)	
**Gender**									
Female	73 (66.4)	584 (64.0)	0.696	14 (87.5)	88 (80.0)	0.709	101 (65.2)	634 (73.6)	0.038
Male	37 (33.6)	329 (36.0)		2 (12.5)	22 (20.0)		54 (34.8)	227 (26.4)	
**Nationality**									
Lebanese	90 (81.8)	769 (84.2)	0.608	13 (81.2)	97 (88.2)	0.428	132 (85.2)	634 (73.6)	0.540
Others	20 (18.2)	144 (15.8)		3 (18.8)	13 (11.8)		23 (14.8)	227 (26.4)	
**Major**									
Arts and Sciences	34 (30.9)	285 (31.2)		2 (12.5)	22 (20.0)		55 (35.5)	269 (31.2)	
Business	30 (27.3)	235 (25.7)		1 (6.25)	3 (2.7)		26 (16.8)	106 (12.3)	
Pharmacy	22 (20.0)	163 (17.9)	0.900	12 (75.0)	73 (66.4)	0.806	42 (27.1)	306 (35.5)	0.194
Engineering	18 (16.4)	160 (17.5)		1 (6.25)	8 (7.3)		18 (11.6)	88 (10.2)	
Education	6 (5.5)	70 (7.7)		0 (0)	4 (3.6)		14 (9.0)	92 (10.7)	
**Marital status**									
Single	102 (92.7)	872 (95.5)		14 (87.5)	105 (95.5)		142 (91.6)	804 (93.4)	
Married	7 (6.4)	34 (3.7)	0.30	1 (6.25)	5 (4.5)	0.09	12 (7.7)	53 (6.2)	0.519
Separated/ Divorced/ Widowed	1 (0.9)	7 (0.8)		1 (6.25)	0 (0.0)		1 (0.6)	4 (0.5)	
**Smoking**									
Current	14 (12.7)	159 (17.4)		3 (18.8)	13 (11.8)		38 (24.5)	197 (22.9)	
Former	8 (7.3)	28 (3.1)	0.055	1 (6.25)	5 (4.5)	0.433	9 (5.8)	37 (4.3)	0.609
Never	88 (80.0)	726 (79.5)		12 (75.0)	92 (83.6)		108 (69.7)	627 (72.8)	
**Alcohol consumption**									
No	104 (94.5)	871 (95.4)	0.874	16 (100)	101 (91.8)	0.602	146 (94.2)	789 (91.6)	0.357
Yes	6 (5.5)	42 (4.6)		0 (0.0)	9 (8.2)		9 (5.8)	72 (8.4)	
**Monthly income**									
<$500	12 (10.9)	82 (9.0)		3 (18.8)	2 (1.8)		12 (7.7)	44 (5.1)	
$500–999	7 (6.4)	99 (10.8)		2 (12.5)	15 (13.6)		26 (16.8)	146 (17.0)	
$1000–1999	12 (10.9)	94 (10.3)	0.232	0 (0.0)	20 (18.2)	0.005	18 (11.6)	111 (12.9)	0.322
≥$2000	11 (10.0)	51 (5.6)		4 (25.0)	14 (12.7)		32 (20.6)	136 (15.8)	
Don’t know/No response	68 (61.8)	587 (64.3)		7 (43.8)	59 (53.6)		67 (43.2)	424 (49.2)	
**Presence of comorbidities**									
No	107 (97.3)	894 (97.9)	0.723	16 (100)	108 (98.2)	1	135 (87.1)	776 (90.1)	0.318
Yes	3 (2.7)	19 (2.1)		0 (0.0)	2 (1.8)		20 (12.9)	85 (9.9)	

**Table 2:** Vaccination characteristics of the study population in 2015–2016, 2017–2018 and 2021–2022 studies.

Characteristics	2015–2016 (N = 1023)	2017–2018 (N = 126)	2021–2022(N = 1016)
	*n* (%)	*n* (%)	*n* (%)
**Received the influenza vaccine this year**			
No	913 (89.2)	110 (87.3)	861 (84.7)
Yes	110 (10.8)	16 (12.7)	155 (15.3)
Place of getting the vaccine? *			
Pharmacy	86 (78.2)	13 (81.3)	33 (21.3)
Physician clinic	18 (16.4)	2 (12.5)	10 (6.45)
Health center	3 (2.7)	0 (0.0)	21 (13.5)
Hospital	1 (0.9)	1 (6.3)	79 (51.0)
Others	2 (1.8)	0 (0.0)	12 (7.7)
Why did you receive the influenza vaccine? *			
Due to the current presence of COVID-19 pandemic	-	-	95 (61.3)
I believe that I am at risk of catching it	-	12 (75.0)	42 (27.1)
I want to reduce the risk of transmitting it to others	-	3 (18.8)	56 (36.1)
I believe that flu vaccine is generally safe	-	5 (31.3)	56 (36.1)
I believe that flu vaccine is effective	-	8 (50.0)	43 (27.7)
I believe flu is a serious disease	-	3 (18.8)	28 (18.1)
Others encouraged me to take it	-	2 (12.5)	19 (12.3)
I have a chronic disease	-	0 (0)	2 (1.3)
I was informed about it through “Beat the Flu” campaign	-	4 (25.0)	17 (11.0)
Reasons for not getting the vaccine **			
I don’t need it	390 (42.7)	45 (40.9)	315 (36.6)
I don’t believe it is beneficial	160 (17.5)	23 (20.9)	134 (15.6)
It costs too much	17 (1.9)	1 (0.9)	54 (6.3)
I don’t like needles	140 (15.3)	27 (24.5)	94 (10.9)
I might get sick/get some side effects	62 (6.8)	10 (9.1)	124 (14.4)
It is not available in my region	20 (2.2)	4 (3.6)	108 (12.5)
A doctor/pharmacist did not recommend it	157 (17.2)	11 (10)	131 (15.2)

* among vaccinated, ** among unvaccinated.

**Table 3:** Effect of COVID-19 on influenza vaccination in the 2021–2022 study.

Characteristics	Vaccinated (N = 155)	Not-Vaccinated (N = 861)	*p*-Value
	*n* (%)	*n* (%)	
**How has COVID-19 changed your attitude toward influenza vaccine?**			
Vaccines are effective and crucial to guaranteeing public health	66 (42.6)	305 (35.4)	0.107
Taking flu vaccine is important in order not to mix flu symptoms with COVID-19 symptoms	50 (32.3)	181 (21.0)	0.0029
Vaccines are important to avoid pandemics	73 (47.1)	320 (37.2)	0.025
Vaccines are a fraud designed to profit pharmaceutical companies	12 (7.7)	52 (6.0)	0.107
My attitude didn’t change	17 (11.0)	305 (35.4)	<0.0001
**Did you receive the COVID-19 vaccine?**			
Yes, one dose	27 (17.4)	75 (8.7)	
Yes, two doses	108 (69.7)	492 (57.1)	<0.0001
Yes, three doses	9 (5.8)	27 (3.1)	
No	11 (7.1)	267 (31.0)	

## Data Availability

The data presented in this study are available on request from the corresponding author.
